# Burden of pain in back and extremities in rural population: A community-based estimation of 12-month prevalence, distribution and duration of pain in rural Gadchiroli, India

**DOI:** 10.7189/jogh.11.12001

**Published:** 2021-11-27

**Authors:** Anand A Bang, Shekhar Y Bhojraj, Mahesh Deshmukh, Vinay R Joshi, Tushar Yarmal, Sameer Kalkotwar, Abhay T Bang

**Affiliations:** 1 Society for Education, Action and Research in Community Health (SEARCH), Gadchiroli, Maharashtra, India; 2 Spine Foundation, Mumbai, Maharashtra, India; 3 Hinduja Hospital and Research Center, Mumbai, Maharashtra, India; 4 Naraindas Morbai Budhrani Trust, Mumbai, Maharashtra, India

## Abstract

**Background:**

Population based estimates of the burden of pain in back and extremities (PBE) are lacking from rural India. We estimated this burden, measured as a) 12-month prevalence, b) site specific prevalence c) total number of painful sites per adult, d) severity of pain and e) duration of pain in the rural adult population in Gadchiroli, India, over a period of 12 months.

**Methods:**

This population-based, cross-sectional study was conducted in two villages randomly selected from a cluster of 7 eligible villages in Gadchiroli district of India. All adults ≥20 years in these villages were surveyed by the trained community health workers in January 2010 by making household visits. The data were collected using a structured, pretested questionnaire on the history of pain in back and extremities (PBE) at various anatomical sites and its features during the previous 12 months.

**Results:**

Out of 2535 eligible adults in two villages, 2259 (89%) were interviewed, of which 1876 (83%) had an episode of PBE in the preceding 12 months. The period prevalence of pain was 76% in back (including lower back, thoracic and neck) and 71% in the extremities. Highest site specific prevalence was at lower back (70%), knee (46%), neck (44%), leg/calf (39%) and mid-back (39%). The mean number of painful sites per adult was 4.57 (standard deviation (SD) = 4.17). The prevalence of severe pain was 15%. The mean number of painful days due to PBE was 166 days. Female gender (odds ratio (OR) = 2.8, 95% confidence interval (CI) = 2.1-3.6), farming/labour occupation (OR = 1.8, 95% CI = 1.4-2.4), increasing age (more than 60 years OR = 6.3, 95% CI = 3.3-11.9) were significantly associated with the risk of PBE.

**Conclusion:**

Nearly five out of six adults in rural Gadchiroli suffered from pain in back or extremities during the preceding 12 months. Pain was at multiple sites and was present on a mean 166 days in the year. Female gender, farming / manual labor as occupation and increasing age were the key risk factors identified. The pain in back and extremities emerges as a public health priority in rural communities.

Back pain (BP) and musculoskeletal pain (MSP) are the commonest form of chronic pain, causing disability and health care expenditure globally [1-4]. Globally, the lifetime prevalence of low back pain (LBP) is around 70%-85%, with 12-month prevalence ranging from 15%-45% and point prevalence around 30% [5]. Although the exact estimates vary across different countries and societies, according to the World Health Organization, “the incidence and prevalence of low back pain are roughly the same the world over – wherever epidemiological data have been gathered or estimates made” [2]. In addition to back pain, musculoskeletal pain is also a significant problem. The Global Burden of Disease Study 2010 [3] estimated that globally MSP caused loss of 2462 Disability Adjusted Life Years (DALYs) per 100 000 population and was ranked as the second highest cause of disability. LBP caused loss of 1206 (822-1264) DALYs per 100000 globally and was ranked as the sixth highest cause of DALYs lost. Two community based studies from rural India reported that the point prevalence of rheumatic musculoskeletal pain is 18.2% and 26% respectively [6,7].

Heavy physical work is a known risk factor for back and musculoskeletal pain [8] and hence agrarian rural communities across the world are at a high risk of BP and MSP [9-11]. The implications of pain and physical suffering coupled with mental distress, disability and economic loss for a population, which mainly depends on manual labor for livelihood, may be severe. Unfortunately, there is a dearth of population-based data from rural agrarian India on the burden of back and musculoskeletal pain, the resulting disability and the diagnostic profile. Such data can guide the development of appropriate public health interventions for BP and MSP.

A non-government organization SEARCH (Society for Education, Action and Research in Community Health), located in Gadchiroli district in India, annually organizes local Health Assemblies involving rural and tribal representatives. Back pain has been identified as a high priority health problem. Hence pain in back and extremities (PBE) was selected for community based research. This study aims to estimate the burden of PBE in adult population (≥20 years) in rural Gadchiroli in the Maharashtra state in India.

## METHODS

### Study setting

The study was conducted in villages in Gadchiroli district which is situated on the eastern border of the Maharashtra state in India (Figure S1 in the **Online Supplementary Document**). Total population of the district is 1 071 795 according to the Census 2011 [12]. An estimated ~ 30% of the district’s population lives below the poverty line making it one of the least developed districts in the state, as well as in India [13]. The Human Development Index (HDI) of Gadchiroli was the lowest in the state at 0.21 as compared to the HDI of 0.58 for the state of Maharashtra and 1 for the city of Mumbai [14]. The main source of livelihood is paddy cultivation and forest produce [13]. The adult literacy rate is 70%. Health care is provided primarily through the public health system comprising of one district hospital, 13 smaller Rural Hospitals for each block, 45 Primary Health Care Centers (PHC), each catering approximately 20 000 to 30 000 population and 376 Sub Health Centers (SHC), each catering to 3000 to 5000 population [13].

The SEARCH has a field research area of 86 villages spread over 3 Tahsils. In these villages, community health workers (CHWs) regularly collect population-based information as part of the demographic surveillance system in place and provide health care for selected ailments to the villagers [15].

### Study design and sample

We conducted a population based, cross-sectional, interview-based study of history of pain in back and extremities during the period of the previous 12 months. The sample size was estimated to measure the primary outcome of period prevalence (assumed 15%) of low back pain in the adults (≥20 years of age), at a desired level of precision of 0.02. The design effect (due to the clustering of the pain in a village) was expected to be 1.25. The estimated sample size (1600) was further increased by 15% to compensate for the non-response rate, making the required sample size to be 1840.

The villages were selected from the field research and service area of SEARCH of 39 villages in the Gadchiroli tehsil. The study villages (two) were selected by a two-stage procedure. First, villages were identified by applying eligibility criteria such as (i) presence of residential and functional male and female CHW of SEARCH in the village to ensure complete data collection and optimum participation by women, (ii) adult (≥20 years) population was >1000, (iii) villages were more than 5 km away from Gadchiroli town and (iv) villages that did not have a hospital or PHC located in it. Villages with larger population (>2000) were excluded to avoid undue weight in the sample. Villages with PHCs as well as villages within 5 km distance from the district headquarter were excluded as usual Indian villages have limited access to such health facilities. Based on these criteria, 7 villages were identified from the 39 villages as eligible forming the sampling frame of the study. From this, the study villages were randomly selected till the required sample size (1840 adults) was met. Two villages, Mudza and Bamhani were thus selected which were 7 and 12 km from the district headquarter respectively and 20 km from each other. In both the villages agriculture was the primary source of livelihood, had roads which were functional in all seasons and had health sub-centers. All the resident adults (≥20 years of age) from these two villages as recorded in the population register of SEARCH were eligible to be included the study.

### Data collection

#### Questionnaire development

A questionnaire in vernacular language (Marathi) was developed to survey the participants and record the following information about the immediate past 12 months from January 2009 to December 2009, a) episodes of pain in back and extremities at different anatomical sites; b) intensity, duration and month of the episode and c) number of painful sites per adult The questionnaire also recorded information on common sociodemographic characteristics including education, which was categorized into 6 categories, from illiterate to education more than 12 years. The questionnaire was pilot tested on 100 individuals in villages that were not part of the study and in the rural clinic of SEARCH and revised accordingly. We classified pain in the back and extremities as acute if the pain duration was up to 42 days (6 weeks), sub-acute if the pain was for more than 6 weeks to 12 weeks and chronic if the pain was for more than 12 weeks [16].

#### Training and quality control

The male and female CHWs (total four) from the two selected study villages and the four field supervisors of SEARCH were trained for three days by a training team consisting of physician (AAB), statistician (MD), and public health researcher (TY) using standard guidelines in administering the questionnaire and recording. The supervisors were trained further for two days in using various checklists for field data quality control and supervision. The CHWs and supervisors were rigorously evaluated and allowed to participate in the study only when they scored >85% in the post training evaluation. Any mistakes noted during evaluation were rectified by re-training the CHWs and supervisors.

#### Data collection

The data were collected from 1 January 2010 to 25 January 2010 about the history of pain in the previous 12-month period from 1 January 2009 to 31 December 2009. Male and female CHWs of SEARCH maintain village population registers which are updated every 6 months, and in which a Unique Identification Number was assigned to every resident. Using these, the list of all the 2535 adult participants (≥20 years) in the two villages was generated. The CHWs visited all the households and after taking informed consent, administered the questionnaire in a face-to-face interview with the eligible participants, usually 20 to 25 per day. Male and female CHWs interviewed the participants of respective gender only. The filled questionnaires were collected every week by the supervisors who checked the quality and completeness of each filled questionnaire using a data quality checklist. Questionnaires with missing information were returned back to the CHWs for collecting the missing information. The supervisors randomly attended on a sample basis the interviews by the CHWs in the first week of data collection and checked the method. The supervisors also randomly visited 5% of the households to verify the data collected by the CHWs. The remuneration of the CHWs was linked with the quality of the data. After completing the interview, the CHWs offered tablet Ibuprofen 400 mg three times a day for seven days, free of cost, to all the participants with pain in back and extremities.

### Statistical methods

A database was constructed for data entry using FOX PRO Version 2.0. The data were double entered and checked for inconsistencies. No statistical adjustments were done in the analysis for the eligible participants who could not be interviewed or the two stage sampling method used for selection of villages. “Student's t-test was used for assessing difference between group means after checking for normality. Differences between percentages were assessed using chi-square test. We used two-sided tests with a significance level of 5%.” The effect of predictor variables on the risk of any pain in back and extremities was assessed by multivariate logistic regression model. We followed STROBE guidelines for the reporting of observational studies. Analyses were conducted using Stata 10.0 (Stata Corp, College Station, Texas, USA).

### Ethical approval

The research followed the tenets of the Declaration of Helsinki. Ethical approval for this nested study was granted as part of the main study, by the Institutional Ethical Committee (IEC) of SEARCH formed according to the guidelines by the Indian Council for Medical Research. The IEC reference number was 20091212-01. Consent was obtained first at the cluster level in the study villages 15 days before starting the survey. The community leaders (Village Council Leaders and members, school teacher and presidents of microfinance self-help groups) were explained the purpose and scope of the study including the benefits to the villagers (availability of referral care in SEARCH clinic and the care through a village clinic). Informed written consent in vernacular language in a standard format was obtained from individual participants after explaining the nature and benefits of the study. The benefits provided during the study included free consultation by spine surgeons and rheumatologists in a clinic conducted in the same village at a later date. For those who needed further evaluation, laboratory investigations, as well as imaging with Magnetic Resonance Imaging (MRI) and x-ray including transport were provided free of cost. For patients needing pharmacotherapy and physiotherapy, these services were also provided free of cost and for those needing surgical interventions, such services were provided at significantly subsidized costs. The CHW discussed these benefits using a printed pamphlet.

## RESULTS

### The study population and its characteristics

**Figure 1:**
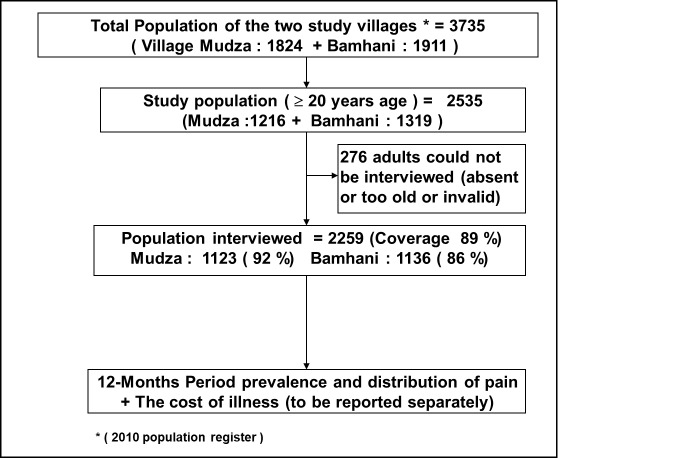
Study design flowchart.

Total population of the two study villages was 3735 out of which 2535 (67.9%) were adults ≥20 years of age and were eligible to participate in the study (**Figure 1**). Of these, 2259 (89%) were interviewed while 276 (11%) were either absent from the village (migrated for work) or unable to communicate due to very old age or disability. The response rate was higher in Mudza (92%) than Bamhani (86%), and for women (91.3%) than men (87.5%). There were no refusals to participate in the study.

The mean age of the participants was 41.7 years (SD = 15.6) and 51% of the study participants were women. The occupation was primarily farming and farm labour (78%). While about 61% of the study participants were literate, only 10% of the participants had schooling beyond 10 years. The age, caste, sex, education and occupation distribution of the participants are presented in **Table 1**.

**Table 1:** Socio-demographic characteristics of the population studied (n = 2259)

Characteristic	Participants	
	**No**	**%**
**Sex**		
Males	1101	48.7
Females	1158	51.3
**Caste:**		
Schedule castes	215	9.5
Schedule tribes	318	14.1
Other castes	1726	76.4
**Education:**		
Illiterate	887	39.3
1-4 y	395	17.5
5-7 y	274	12.1
8-10 y	472	20.9
11-12 y	190	8.4
>2 y	41	1.8
**Age (years):**		
20-30	678	30.0
31-40	516	22.8
41-50	471	20.8
51-60	301	13.3
>60	293	13.0
Mean age (SD)	41.7 (15.6)	
**Occupation:**		
Labour	999	44.2
Farmer	765	33.9
Salaried jobs	69	3.1
Household work	193	8.5
Business	157	6.9
Other	76	3.4

SD – standard deviation, y – years

### 12-month prevalence of pain in back and extremities

**Figure 2:**
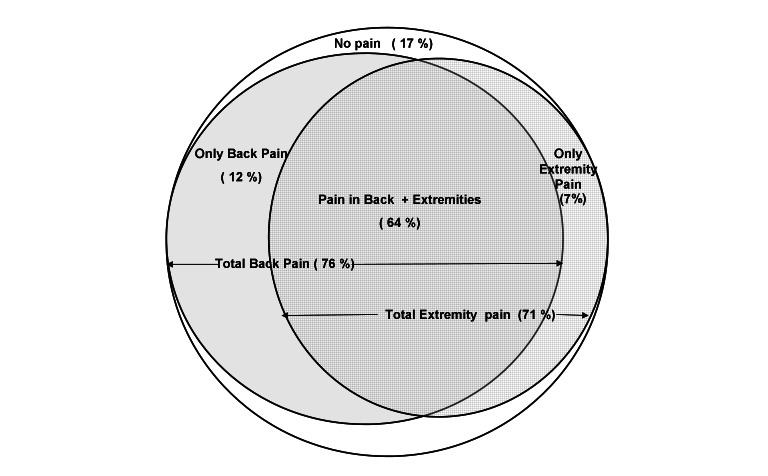
Period prevalence of pain in back and extremities in rural Gadchiroli.

Of the 2259 individuals surveyed, 1876 had an episode of pain in the back and/or extremities in the twelve months preceding the survey, giving an overall period prevalence of 83% (95% CI = 81.4-84.6) presented in **Figure 2**. Overall, 76% (95% CI = 74.2-77.8) of the participants had back pain (including lower back, thoracic and neck) and 71% (95% CI = 69.0-72.8) had pain in extremities. The prevalence of only back pain (12%) and pain only in the extremities (7%) was significantly less compared to the prevalence of pain in both back and extremities (64%). 17% of the participants did not suffer any pain (**Figure 2**).

### 12-month prevalence by the anatomical site

**Figure 3:**
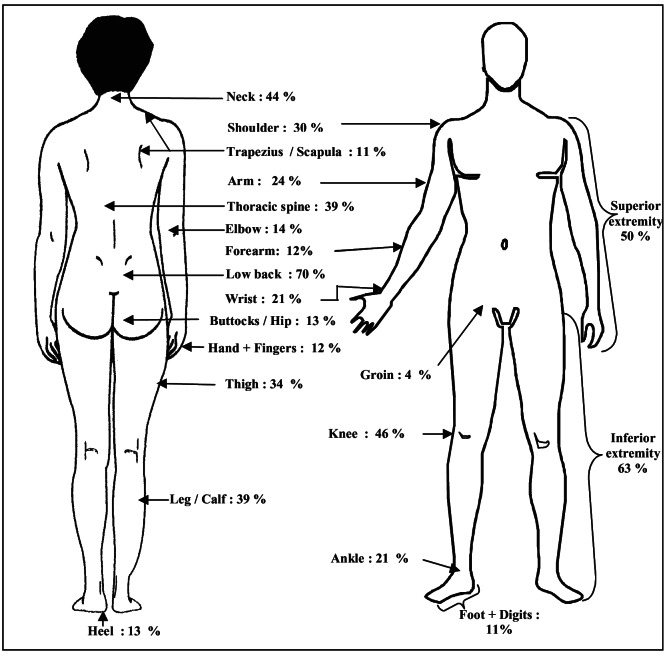
Period prevalence of pain in back & extremities at different sites.

The period prevalence of pain at various anatomical sites is presented in **Figure 3** and Table S1 in the **Online Supplementary Document**. The highest site specific period prevalence of pain was at low back (70%). The pain in the inferior extremity (63%) was more prevalent than in the superior extremity (50%), knee (46%) and neck (44%). The site with the lowest prevalence of pain was groin (4%).

### Duration of pain

Individuals suffered pain in back and/or extremity for a significant period in the year (Table S2 in the **Online Supplementary Document**). In the symptomatic adults (n = 1876), the mean duration of back pain was 171 days (SD = 152, 95% CI = 164.6-178.4), of the pain in extremity was 144 days (SD = 150, 95% CI = 137.2-150.8) while any (back and/or extremity) pain was present on 200 days (SD = 149, 95% CI = 193.5-207.0). In the total population studied (n = 2259) including the asymptomatic adults, an adult ≥20 years of age suffered from back pain for 142 days (SD = 153, 95% CI = 136.1-148.7), extremity pain for 120 days (SD = 147, 95% CI = 113.5-125.7) and back and/or extremity pain for 166 days (SD = 155, 95% CI = 159.9-172.7). Overall, in the sample population, pain in the inferior extremity was present for significantly more number of days (101 days, SD = 140, 95% CI = 95.5-107.1) than the superior extremity (73 days, SD = 127, 95% CI = 67.4-77.9).

The 12-month prevalence of acute pain (≤6 weeks) anywhere was about 20%, of subacute pain (6-12 weeks) much less (8%) while that of chronic pain (>12 weeks) at these sites was around 55%. The prevalence of acute pain was similar for neck, lower back, inferior extremity and superior extremity (22%, 20%, 21% and 21% respectively) but lower in the mid-back (14%). The pain in the inferior extremity was more prone to be chronic (35%) than the pain in the superior extremities (24%) as was the pain in low back (44%) compared to neck (18%) and thoracic (21%).

### Number of painful sites per adult

**Figure 4:**
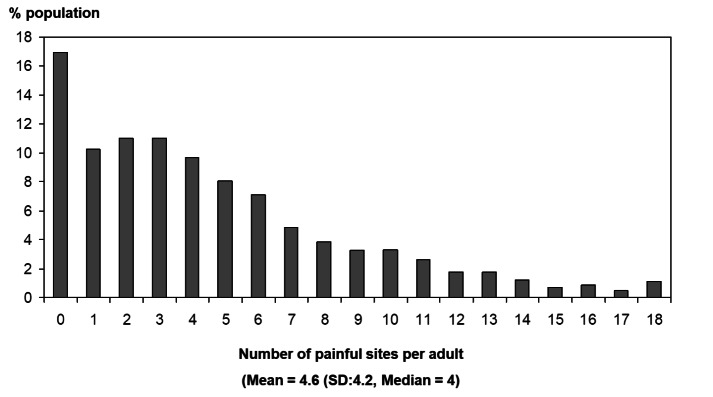
Number of painful sites per adult in rural population in Gadchiroli.

The number of painful sites per adult in the population studied is presented in **Figure 4** and Table S3 in the **Online Supplementary Document**. The mean number of painful sites was 4.57 (SD: 4.17). As many as 50.1% of study participants had pain at 1-5 sites, 22.4% participants had pain at 6-10 sites while 10.5% participants had pain at >10 sites.

### Intensity of pain

**Figure 5:**
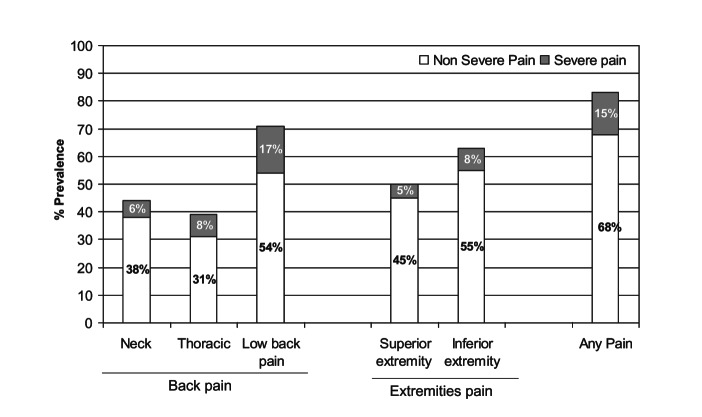
Intensity of pain according to anatomical sites.

Majority of the symptomatic adults had a non-severe pain. The prevalence of non-severe pain was 68%, 62%, 54% and 58% for any pain, any extremity pain, low back and any back respectively (**Figure 5**, Table S4 in the **Online Supplementary Document**). In case of low back pain, the percentage prevalence of severe pain (17%) was substantially higher than at other anatomical sites.

### Predictors of any pain in back or extremities

In considering the various predictors of any pain in back and extremities, we focused on key categories of gender, education, age and occupation (**Table 2**). We found that women had significantly more risk of PBE (OR = 2.6, 95% CI = 2.1-3.6) than men. There was a gradient in reduced risk of PBE with increasing education, with OR of 1 for illiterate to 0.3 (95% CI = 0.1-0.5) in case of education more than 12 years. On the other hand, there was a steep increasing gradient of risk of PBE with increasing age, with OR of 1 for the reference age group of 20 to 30 to OR of 6.3 (95% CI = 3.3-11.9) for age >60. Finally, occupation of farmer/laborer had significantly more risk of PBE (OR = 1.8, 95% CI = 1.4-2.4) in comparison to any other occupation.

**Table 2:** Risk factors associated with any pain in back or extremities (n = 2259)

Predictor	Odds ratio	95% CI
**Sex:**		
Males	1	
Females	2.8	(2.1, 3.6)
**Education:**		
Illiterate	1	
1-4 y	0.8	(0.5, 1.2)
5-7 y	0.6	(0.4, 0.9)
8-10 y	0.4	(0.2, 0.5)
11-12 y	0.4	(0.2, 0.7)
>12 y	0.3	(0.1, 0.5)
**Age (years):**		
20-30	1	
31-40	1.6	(1.2, 2.2)
41-50	2.3	(1.5, 3.3)
51-60	3.6	(2.1, 6.1)
>60	6.3	(3.3, 11.9)
**Occupation:**		
Other	1	
Labour/farmer	1.8	(1.4, 2.4)

CI – confidence interval, y – years

## DISCUSSION

We found a very high burden of pain in back and extremities in the rural community in Gadchiroli. The 12-month prevalence of any pain (83%), back pain (76%) as well as pain in extremities (71%) was very high. Only 17% of the adults were free of back musculoskeletal pain in a year. The pain was at multiple sites; of prolonged duration with an average adult experiencing pain at some site for 166 days in a year as well as chronic in nature for 55% of the participants. Majority of the adults (68%) had pain of mild or moderate intensity.

Anatomically, the load bearing sites experienced higher prevalence of pain and of longer duration. The prevalence was highest in low back (70%), followed by inferior extremity as a whole (63%), knee (46%) and neck (44%). Similarly, the mean number of days of pain per adult was 129 (SD = 150) in low back compared to 54 (SD = 111) in neck, or 101 (SD = 140) days in inferior extremity compared to 73 days in superior extremity. The high prevalence of low back pain in women could be partly due to the bent position women adopt for long hours during planting saplings of paddy and harvesting and in men due to the heavy load carried by them. The high prevalence of neck pain can be attributed to the practice of carrying heavy load (firewood, earth) on head for long distance, especially by women.

The mean duration of any pain in a year (166 days) and the higher prevalence of chronic pain (55%) indicate the predominantly chronic nature of the problem. It could also suggest the lack of availability of pain relief treatment in the community. The multiple painful sites (mean 4.6) suggest that the problem is not a localized one, and possibly caused by the widespread wear and tear due to manual labour. Interestingly, majority of the participants said that the pain was less than severe.

The higher risk of PBE in women in this study was similar to studies from rural China [8], Tanzania [17], National Capital Region of India [18] but different from others [19,20] which found either no gender specific difference or higher burden in men. The reason for higher burden in women in our study could be repetitive physical work or physiological, of having a weaker bone frame though we do not have conclusive evidence of the same including serum calcium levels. The higher risk associated with farming and labour as an occupation probably corroborates with other studies from rural China [8], Nigeria [20,21] and Lesotho [22]. The possible pathway could be repetitive mechanical stress on the body arising from rigorous physical labour. Alternatively, it may be also due to relatively less intake of proteins and muscle mass in the body which needs to be further studied through dietary and ergonomic studies. This pathway may also be functional in the effects of increasing age on increasing the risk of PBE despite possibly less engagement of elderly people in hard manual labour.

Education is inversely proportional to the risk of PBE is probably due to change in the occupation, from farming and agricultural labour to more while collar occupation involving less physical labour.

There are very few studies that have examined the period prevalence of pain in developing countries. The 12-month prevalence of back pain in this rural population (76%) was higher than some other studies conducted in rural Tibet (41.9%) [19], rural China (64.1%) [20] and similar to found in the farmers in Nigeria (72.4%) [20]. The possible reasons for the higher prevalence in our study could be the heavy physical work and the relative lack of availability of care.

To the best of our knowledge, this is the first study from rural India comprehensively reporting population based period prevalence of pain in back and extremities including the site specific prevalence, the number of painful sites per adult and the duration of pain in a community. The participants in this study are more representative of the agrarian Indian population, with higher dependence on manual labour. Other studies were either restricted to a certain type of labour population only, such as drill worker, cashew worker or jute worker [23-25], or were not population based [26]. The finding of higher prevalence of low back pain than knee pain was similar to most of the COPCORD series results and contrary to the findings of the Indian COPCORD series [10,27]. Nevertheless, we suggest that more population-based studies should be conducted in different parts of rural India, possibly employing larger sample sizes, to identify the regional estimates and any differences in the prevalence and profile of pain.

Certain strengths of the study lend confidence to the estimates obtained. The two study villages were randomly selected. The participation rate of the adults in the villages was high (89%). The socioeconomic and demographic characteristics of the participants (**Table 1**) do not reveal any serious bias. The data collection was done by CHWs with more than 15 years of experience and was done using a well-tested, structured and robust questionnaire with rigorous quality control.

Three limitations of our study are 1) the small number of sampled villages which did not include a large variety by the size or the availability of health care. 2) The possibility of recall loss in reporting of pains from preceding 12 months which may have underestimated the period prevalence. 3) The study villages had the presence of the male and female CHWs who routinely provided subsidized treatment for pains with tablet aspirin for the past 20 years. This may have reduced the period prevalence of pains by reducing the duration of pain.

Overall, the high burden of PBE observed in this study corroborates with the perception voiced by the community representatives in the Health Assembly that this is a priority health problem in rural Gadchiroli. However, it is not considered a public health priority currently in India. This could be due to lack of population-based data on PBE as well as due to the non-fatal nature of the problem. Given the significant activity limitation associated with pain in back and extremities in our study [28] as well as in other studies [2,29], any chronic diseases care program for rural population needs to be broadened to include pain management. Considering the non-severe nature of the pain in majority of the participants, such an intervention has the potential to provide substantial relief.

## CONCLUSION

This population-based study in agrarian rural community in Gadchiroli found a high burden of pain in back and extremities. The pain was at multiple sites in body and for a mean 166 days in a year resulting in chronic suffering. Female gender, increasing age and manual labour including farming were significantly associated with the risk of PBE. The pain in back and extremities emerges as a public health priority in rural communities. Systematic epidemiological studies need to be conducted to understand the burden of this problem in the other part of rural India as well as risk factors including causative pathway. Any chronic non-communicable disease control program should include the component of pain alleviation and management of musculoskeletal pain in back and extremities.

## Additional material


Online Supplementary Document

## References

[1] Woolf AD The Bone and Joint Decade 2000-2010. Ann Rheum Dis. 2000;59:81-2. 10.1136/ard.59.2.81 10666159 PMC1753078

[2] Ehrlich GE Low back pain. Bull World Health Organ. 2003;81:671-6. 14710509 PMC2572532

[3] Murray CJL Vos T Lozano R Naghavi M Flaxman AD Michaud C Disability-adjusted life years (DALYs) for 291 diseases and injuries in 21 regions, 1990–2010: a systematic analysis for the Global Burden of Disease Study 2010. Lancet. 2012;380:2197-223. 10.1016/S0140-6736(12)61689-4 23245608

[4] Campbell C Muncer SJ The causes of low back pain: a network analysis. Soc Sci Med. 2005;60:409-19. 10.1016/j.socscimed.2004.05.013 15522495

[5] Andersson GB Epidemiological features of chronic low-back pain. Lancet. 1999;354:581-5. 10.1016/S0140-6736(99)01312-4 10470716

[6] Chopra A Saluja M Patil J Tandale HS Pain and disability, perceptions and beliefs of a rural Indian population: A WHO-ILAR COPCORD study. WHO-International League of Associations for Rheumatology. Community Oriented Program for Control of Rheumatic Diseases. J Rheumatol. 2002;29:614-21. 11908580

[7] Mathew AJ Chopra A Thekkemuriyil DV George E Goyal V Nair JB Impact of musculoskeletal pain on physical function and health-related quality of life in a rural community in south India: a WHO-ILAR-COPCORD-BJD India study. Clin Rheumatol. 2011;30:1491-7. 10.1007/s10067-011-1824-0 21853278

[8] Barrero LH Hsu Y-H Terwedow H Perry MJ Dennerlein JT Brain JD Prevalence and Physical Determinants of Low Back Pain in a Rural Chinese Population. Spine. 2006;31:2728-34. 10.1097/01.brs.0000244583.35982.ea 17077743

[9] Louw QA Morris LD Grimmer-Somers K The prevalence of low back pain in Africa: a systematic review. BMC Musculoskelet Disord. 2007;8:105. 10.1186/1471-2474-8-105 17976240 PMC2198912

[10] Chopra A Abdel-Nasser A Epidemiology of rheumatic musculoskeletal disorders in the developing world. Best Pract Res Clin Rheumatol. 2008;22:583-604. 10.1016/j.berh.2008.07.001 18783739

[11] Haq SA Darmawan J Islam MN Uddin MZ Das BB Rahman F Prevalence of rheumatic diseases and associated outcomes in rural and urban communities in Bangladesh: a COPCORD study. J Rheumatol. 2005;32:348-53. 15693098

[12] Government of India. Census of India. 2011. Available: http://www.censusindia.gov.in/2011-prov-results/paper2/data_files/mah/8-POP-11-26.pdf. Accessed: 13 August 2013.

[13] Government of Maharashtra. District Collectorate, Gadchiroli. Available: http://gadchiroli.nic.in/. Accessed: 17 January 2012.

[14] Government of Maharashtra. Human Development Report Maharashtra. 2002. Available: http://planningcommission.nic.in/plans/stateplan/sdr_pdf/shdr_maha02.pdf. Accessed: 17 January 2012.

[15] SEARCH. SEARCH, Gadchiroli. Available: http://www.searchgadchiroli.org/. Accessed: 17 January 2012.

[16] Burton AK Balagué F Cardon G Eriksen HR Henrotin Y Lahad A Chapter 2. European guidelines for prevention in low back pain: November 2004. Eur Spine J. 2006;15 Suppl 2:S136-168. 10.1007/s00586-006-1070-3 16550446 PMC3454541

[17] Stelzhammer B. Community-based prevalences of headache and backache in a rural Tanzanian community –epidemiology and characteristics. Thesis for Doctor medicinae universae at Medical University Innsbruck. 2005.

[18] Bihari V Kesavachandran C Pangtey B Srivastava A Mathur N Musculoskeletal pain and its associated risk factors in residents of national capital region. Indian J Occup Environ Med. 2011;15:59. 10.4103/0019-5278.90375 22223951 PMC3249791

[19] Hoy D Toole MJ Morgan D Morgan C Low back pain in rural Tibet. Lancet. 2003;361:225-6. 10.1016/S0140-6736(03)12254-4 12547548

[20] Fabunmi AA Aba SO Odunaiya NA Prevalence of low back pain among peasant farmers in a rural community in South West Nigeria. Afr J Med Med Sci. 2005;34:259-62. 16749358

[21] Omokhodion FO Low back pain in a rural community in South West Nigeria. West Afr J Med. 2002;21:87-90. 12403024

[22] Worku Z Prevalence of low-back pain in Lesotho mothers. J Manipulative Physiol Ther. 2000;23:147-54. 10.1016/S0161-4754(00)90243-4 10771498

[23] Tiwari RR Saha A An epidemiological study of low back pain among oil drilling workers in India. Toxicol Ind Health. 2014;30:60-3. 10.1177/0748233712451771 22740620

[24] Girish N Ramachandra J Arun GNM Asha K Prevalence of musculoskeletal disorders among cashew factory workers. Arch Environ Occup Health. 2012;67:37-42. 10.1177/0748233712451771 22315934

[25] Sett M Sahu S Study on work load and work-related musculoskeletal disorders amongst male jute mill workers of West Bengal, India. Work. 2012;42:289-97. 10.3233/WOR-2012-1352 22699196

[26] Sharma SC Singh R Sharma AK Mittal R Incidence of low back pain in workage adults in rural North India. Indian J Med Sci. 2003;57:145-7. 14510345

[27] Chopra A Patil J Billempelly V Relwani J Tandle HS Prevalence of rheumatic diseases in a rural population in western India: a WHO-ILAR COPCORD Study. J Assoc Physicians India. 2001;49:240-6. 11225138

[28] Bang AA Bhojraj SY Deshmukh M Kalkotwar S Joshi VR Yamal T Activity limitation and disability due to pain in back and extremities in rural population: A community-based study during a period of twelve months in rural Gadchiroli, India. J Glob Health. 2021;11:12003. 10.36076/ppj.2000/3/167 PMC8645241 34912552

[29] Manchikanti L Epidemiology of low back pain. Pain Physician. 2000;3:167-92. 10.36076/ppj.2000/3/167 16906196

