# Supplementary material for: Burden of pain in back and extremities in rural population: A community-based estimation of 12-month prevalence, distribution and duration of pain in rural Gadchiroli, India
Source: J Glob Health. 2021 Nov 27;11:12001. doi: 10.7189/jogh.11.12001 (PMC8645243; doi:10.7189/jogh.11.12001)
Supplement: Online Supplementary Document [file jogh-11-12001-s001.pdf]

Figure S1. Geographical location of Gadchiroli in India

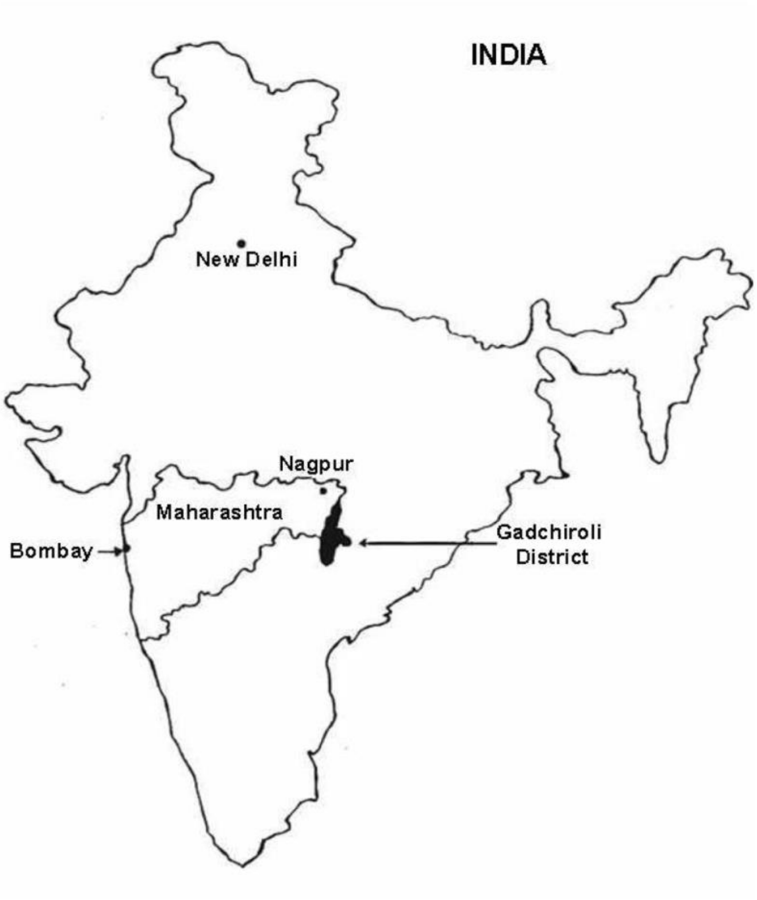

Panel S1. Definitions of anatomical sites where pain was inquired

1. Neck – Posterior (Hind) Part of the body between
    a. External Occipital Protuberance
    b. Most prominent Spine of the Vertebrae (T1)
2. Shoulder – Joint connecting the Trunk and Upper Limb.
3. Arm - Part of Superior Extremity between Shoulder and Elbow
4. Elbow – Joint between Arm and Forearm
5. Forearm – Part of Superior Extremity between Elbow and Wrist
6. Wrist – Joint between Forearm and Hand.
7. Hand and Fingers – Part of the Superior Extremity beyond Wrist.
8. Clavicular region – Part of the body connecting the Neck and the Shoulder.
9. Superior Trapezius area – Part of the body over the Scapular blades.
10. Mid back – Part of the back (Spine) between the region of Neck and Low Back
11. Low back – Part of the Back (Hind) as follows,
    i. Upper border – Level of lower costal margin.
    ii. Lower border – (Lower Gluteal folds)
12. Gluteal / SI – Region of Buttock.
13. Hip – Trochanteric and groin pain
14. Thig
    h – Part of the Inferior extremity between Buttock/Groin and Knee
15. Knee – Joint between the Thigh and Leg
16. Leg – Part of the inferior extremity between the Knee and Ankle
17. Ankle – Joint between Leg and Foot
18. Heel – Dorsum of Hind Foot
19. Rest foot – Part of the Foot beyond Ankle region excluding Heel.

Table S1. Prevalence of pain in back & extremities by the anatomical site (Period January 2009 - January 2010, n=2259)

Site*	No	% Prevalence	95 % CI
**A) Back Pain**	1717	76.0	(74.2, 77.8)
**Neck**	985	43.6	(41.5, 45.7)
**Thoracic**	879	38.9	(36.9, 41.0)
**Low back**	1585	70.2	(68.2, 72.0)
**B) Extremity pains**	1602	70.9	(69.0, 72.8)
**1) Superior Extremity** †	1124	49.8	(47.7, 51.8)
**Trapezius / Scapula**	255	11.3	(10.0, 12.7)
**Shoulder**	677	30.0	(28.1, 31.9)
**Arm**	547	24.2	(22.5, 26.0)
**Elbow**	324	14.3	(12.9, 15.9)
**Forearm**	261	11.6	(10.3, 12.9)
**Wrist**	468	20.7	(19.1, 22.4)
**Hand + Fingers**	277	12.3	(10.9, 13.7)
**Entire Superior Extermity**	46	2.0	(1.5, 2.7)
**2) Inferior Extremity** ‡	1417	62.7	(60.7, 64.7)
**Buttocks / Hip**	286	12.7	(11.3, 14.1)
**Groin**	82	3.6	(2.9, 4.5)
**Thigh**	775	34.3	(32.3, 36.3)
**Knee**	1038	45.9	(43.9, 48.0)
**Leg / Calf**	881	39.0	(37.0, 41.0)

**Ankle**	478	21.2	(19.5, 22.9)
**Heel**	295	13.1	(11.7, 14.5)
**Foot + Digits**	240	10.6	(9.4, 12.0)
**Entire inferior Extermity**	40	1.8	(1.3, 2.4)
**C) Back and extremity pain both**	1443	63.9	(61.9, 65.9)
**D) Only back pain**	274	12.1	(10.8, 13.5)
**E) Only limb pain**	159	7.0	(6.0, 8.2)
**F) Any pain (Back / Extremity)**	1876	83.0	(81.4, 84.6)

* categories are multiple and are overlapping
† (Any one of Shoulder, Arm, Elbow, Forearm, Wrist, Hand + Fingers ,Trapezius / Scapula)
‡ (Any one of Hip/Buttocks, Groin, Thigh, Knee, Leg / calf, Ankle, Heel, Foot + digits)

Table S2. Number of days of pain in various anatomical regions (Period January 2009 - January 2010, n=2259)

Anatomical region *	Mean number of days with pain per symptomatic adult (n=1876)	SD	(95 % CI)	Mean number of days with pain per adult in the study population (n=2259)	SD	(95 % CI)	% prevalence		
**Any back pain**	171.5	152.0	(164.6, 178.4)	142.4	152.8	(136.1, 148.7)	21.3	7.1	47.6
**Neck**	65.7	118.7	(59.4, 70.1)	53.8	110.9	(49.2, 58.3)	22.1	3.8	17.7
**Thoracic**	75.8	129.1	(69.9, 81.6)	62.9	121.0	(57.9, 67.9)	13.5	4.6	20.8
**Low back**	155.7	151.9	(148.8, 162.5)	129.3	150.2	(123.1, 135.5)	19.6	7.0	43.6
**Any extremity pain**	144.0	149.9	(137.2, 150.8)	119.6	146.9	(113.5, 125.7)	22.8	7.5	40.5
**Superior Extremity†**	87.5	135.0	(81.4, 93.6)	72.7	127.4	(67.4, 77.9)	21.1	5.0	23.6
**Inferior Extremity ‡**	122.0	145.0	(115.4, 128.6)	101.3	139.8	(95.9, 107.1)	20.6	7.3	34.7
**Any pain (Back / Extremity)§**	200.2	149.4	(193.5, 207.0)	166.3	155.5	(159.9, 172.7)	20.1	7.8	5.1

* categories are multiple and are overlapping

† (Any one of Shoulder, Arm, Elbow, Forearm, Wrist, Hand + Fingers ,Trapezius / Scapula)

‡ (Any one of Hip/Buttocks, Groin, Thigh, Knee, Leg / calf, Ankle, Heel, Foot + digits)

§ Mean of the longest duration of painful sites

Table S3. Number of painful sites per adult (January 2009 - January 2010, n=2259)

Number of painful sites	n	%
0	383	17.0
1	232	10.3
2	249	11.0
3	249	11.0
4	219	9.7
5	182	8.1
1 to 5	1131	50.1
6	161	7.1
7	110	4.9
8	87	3.9
9	74	3.3
10	75	3.3
6 to 10	507	22.4
>10	238	10.5
Mean number of sites (SD)	4.57 (4.17)	
Total	2259	100.0

Table S4. Intensity of pain at different anatomical sites (n=2259)

Site *	Total	Mild			Severe		
	Number	No	% prevalence	95% CI	No	% prevalence	95% CI
**Back Pain**	1717	1321	58.5	(56.4, 60.5)	396	17.5	(16.0, 19.2)
**Neck**	985	859	38.0	(36.0, 40.1)	126	5.6	(4.7, 6.6)
**Thoracic**	879	703	31.1	(29.2, 33.1)	176	7.8	(6.7, 9.0)
**Low back**	1585	1210	53.6	(51.5, 55.6)	375	16.6	(15.1, 18.2)
**Limb pain**	1602	1397	61.8	(59.8, 63.9)	205	9.1	(7.9, 10.3)
**Superior Extremity**†	1124	1022	45.2	(43.2, 47.3)	102	4.5	(3.7, 5.5)
**Inferior Extremity**‡	1417	1234	54.6	(52.5, 56.7)	183	8.1	(7.0, 9.3)
**Any pain (Back/limb)**	1876	1529	67.7	(65.7, 69.6)	347	15.4	(13.9, 16.9)

* categories are multiple and not exclusive

† (Any 1 of Shoulder, Arm, Elbow, Forearm, Wrist, Hand + Fingers ,Trapezius / Scapula)

‡ (Any 1 of Hip/Buttocks, Groin, Thigh, Knee, Leg / calf, Ankle, Heel, Foot + digits)
